# PD‐L1, PD‐1, and CTLA‐4 mRNA In Situ Expression by Canine Oral Melanoma Cells and Immune Cells of the Tumour Microenvironment

**DOI:** 10.1111/vco.13039

**Published:** 2025-01-09

**Authors:** Greta Foiani, Erica Melchiotti, Katia Capello, Ilaria Porcellato, Chiara Brachelente, Selina Iussich, Davide Giacobino, Emanuela Morello, Marina Martano, Paolo Buracco, Marta Vascellari

**Affiliations:** ^1^ Histopathology Laboratory Istituto Zooprofilattico Sperimentale delle Venezie Padua Italy; ^2^ Epidemiology and Biostatistics Unit Istituto Zooprofilattico Sperimentale delle Venezie Padua Italy; ^3^ Department of Veterinary Medicine University of Perugia Perugia Italy; ^4^ Department of Veterinary Sciences University of Turin Grugliasco Italy; ^5^ Department of Veterinary Medical Sciences University of Parma Parma Italy

**Keywords:** CTLA‐4, dog, immune checkpoints, melanoma, PD‐1, PD‐L1

## Abstract

Canine oral melanoma (OM) exhibits poor prognosis and limited treatment options. The success of immune checkpoint inhibitors (ICIs) in human melanoma has driven interest in similar therapeutic approaches in the dog, although the immunosuppressive mechanisms adopted by canine OM remain unclear. This study aimed to evaluate the expression of the immune checkpoints PD‐1/PD‐L1 and CTLA‐4 by RNAscope in situ hybridization (ISH) in canine OM, to investigate their expression pattern and explore their potential role in melanoma progression. Twenty‐four formalin‐fixed, paraffin‐embedded canine OM were included in the study. PD‐L1 expression by tumour cells was detected in 100% melanomas (score 1–3), especially at the host‐tumour interface. PD‐1 and CTLA‐4 expression by tumour cells was detected in 13/24 (54%, score 1–2) and 18/24 (75%, score 1) melanomas, respectively. Dual ISH‐immunohistochemistry with Melanoma Triple Cocktail, CD3, CD20 and Iba1 demonstrated the expression of tested immune checkpoints in neoplastic and immune cells. Notably, PD‐1 and CTLA‐4 were predominantly expressed by tumour‐infiltrating T lymphocytes, while PD‐L1 was primarily expressed by tumour‐associated macrophages. PD‐1 expression in neoplastic cells was significantly correlated with mitotic count (*p* < 0.05), while no associations were found between immune checkpoint expression and disease‐free interval or overall survival. Whole tumour PD‐L1 and PD‐1 expression, assessed by image analysis, correlated to PD‐L1 scores in neoplastic cells and the grade of tumour‐infiltrating lymphocytes, respectively. Collectively, PD‐L1, PD‐1 and CTLA‐4 likely contribute to immunosuppression in canine OM. Further studies are warranted to investigate whether ISH can serve as a biomarker for selecting patients suitable for ICI treatment.

## 1 Introduction

Oral melanoma (OM) is common in dogs and has a highly aggressive behaviour, with high rates of local recurrence, metastasis and poor prognosis [1, 2]. Although rare in humans, mucosal melanoma shares several clinico‐pathological features with its canine counterpart, and research on spontaneous canine tumours offers a valuable opportunity to advance the understanding of the human disease [3, 4].

Tumour cells can escape or impair the host anti‐tumour immunity by several mechanisms, including the exploitation of immunomodulatory systems that normally prevent autoimmunity, known as “immune checkpoints” [5]. These include inhibitory receptors, such as the programmed cell death protein 1 (PD‐1) and the cytotoxic T‐lymphocyte‐associated antigen 4 (CTLA‐4). PD‐1 is expressed by activated T cells and other immune cells within the tumour microenvironment (TME) [6]. The activation of PD‐1 receptor by its ligands inhibits T lymphocyte proliferation, survival and effector functions and induces apoptosis of antigen‐specific T cells in peripheral tissues [6]. One of the PD‐1 ligands, the programmed cell death ligand 1 (PD‐L1), is commonly upregulated in tumour cells, thus blocking the local antitumour T cell response [5]. Moreover, chronic antigen exposure in cancer can lead to high levels of persistent PD‐1 expression, inducing a state of “exhaustion” of antigen‐specific T cells [6].

CTLA‐4, primarily expressed by T cells, dampens the amplitude of early T cell activation stages, especially in secondary lymphoid organs such as lymph nodes [5]. It exerts its functions by counteracting the CD28 costimulatory receptor, with which it shares the ligands CD80 and CD86 on the surface of antigen‐presenting cells [7]. CTLA‐4 also enhances the immunosuppressive activity of regulatory T cells (Tregs) and is upregulated in exhausted T cells [7].

Immunotherapy with antibodies blocking the PD‐1/PD‐L1 axis and CTLA‐4 can restore and improve the anti‐tumour T cell‐mediated immunity [5]. Immune checkpoint inhibitors (ICIs) mediate durable tumour regression for various human cancers [5]. The combination of anti‐CTLA‐4 ipilimumab and anti‐PD1 nivolumab has proven the most effective protocol for advanced cutaneous melanoma [8]. However, long‐term benefit are seen in only approximately 50% of patients, with lower response rate in mucosal melanoma [8, 9]. Immunohistochemical expression of PD‐L1 in cutaneous melanomas, relying on approved reagents, protocols and standardised scoring systems, has been found to correlate with clinical responses to ICIs [10].

The exploration of the utility of immunotherapy strategies in canine melanoma is in its infancy. Recent studies have investigated vaccines, adoptive cell transfer and monoclonal antibodies targeting canine PD‐1/PD‐L1 and CTLA‐4 for OM in dogs [11, 12, 13, 14, 15, 16, 17, 18]. However, the immunosuppressive strategies adopted by canine tumours are poorly characterised, and there is limited availability of diagnostic reagents and immunotherapeutic agents in this species [2]. Overexpression of PD‐L1 protein on tumour cells and PD‐1 on tumour infiltrating lymphocytes (TILs) has been observed in several canine cancers, including oral and cutaneous melanoma, using both IHC and immunofluorescence (IF) [19, 20, 21]. In canine cutaneous and oral melanocytic tumours, increased mRNA levels and immunohistochemical expression of CTLA‐4 in lymphocytes have been reported to be associated with a worsened prognosis [22].

The main purposes of this study were to assess the expression of PD‐L1, PD‐1 and CTLA‐4 in canine OM by in situ hybridization (ISH), to analyse the spatial distribution of these molecules within tumour cells and the TME, providing insights into immunosuppressive interactions, and to evaluate potential correlations with histological parameters and disease outcome.

## 2 Material and Methods

### 2.1 Case Selection

Formalin‐fixed paraffin‐embedded (FFPE) samples were obtained from the histopathological archives of the Department of Veterinary Medicine of the University of Turin and Perugia (Italy). Inclusion criteria were the histological diagnosis of OM and availability of FFPE material.

Signalment, clinical (tumour location, staging, therapy) and follow‐up information were collected. The overall survival (OS) was defined as the time from the first diagnosis to the time of death or the end of the study; the disease‐free interval (DFI) was the time from the first diagnosis to the first evidence of disease progression (local recurrence or metastasis).

### 2.2 Histology and Immunohistochemistry

Histological sections were reviewed by three pathologists (G.F., S.I., I.P.) and the following morphological parameters [1] were evaluated:

- Cell morphology (polygonal; spindle; round; mixed);
- Pigmentation (%) [23];
- Mitotic count (number of mitoses in 2.37 mm^2^);
- Nuclear atypia (%);
- Necrosis (%);
- Lymphovascular invasion.

For each case, 4‐μm FFPE tissue serial sections underwent IHC using anti‐Ki67 (1:50; Clone MIB‐1, code M7240, Dako, Glostrup, Denmark), anti‐CD3 (1:200; polyclonal, code A0452; Dako) and anti‐CD20 (1:100, polyclonal, code RB‐9013; Thermo Scientific, Fremont, California) antibodies, for the assessment of the proliferation index and TILs, respectively. IHC for Ki67 was performed on the automated Discovery ULTRA system (Ventana Medical Systems Inc., Roche, Tucson, AZ, USA). For highly pigmented tumours, slides were bleached with a 10% H_2_O_2_ solution in PBS at 42°C for 42 min. Antigen retrieval was performed with Discovery CC1 (pH 8.4, Ventana Medical Systems Inc.) at 95°C for 56 min. Slides were incubated with the primary antibody for 1 h RT. Detection was performed with Discovery Omnimap anti‐mouse HRP and ChromoMap‐DAB (Ventana Medical Systems Inc.). Normal canine lymph node was used as a positive control. Negative controls were performed by omitting the primary antibody. IHC for CD3 and CD20 were performed manually, as previously described [24].

The mean number of Ki67‐positive tumour cells was counted in 1 cm^2^ optical grid reticle in 5 high‐power fields (HPF, 400×) [23]. The mean count of CD3 and CD20‐positive TILs in 5 consecutive HPF was recorded [24]. A previously described four‐level grading system for TILs [24], was applied (grade 0–3).

### 2.3 RNAscope ISH

RNAscope ISH assay was performed in the Discovery ULTRA system (Ventana Medical System Inc.) according to the manufacturer's instructions. The following canine‐specific RNAscope 2.5 VS probes (Advanced Cell Diagnostics [ACD] Inc., Santa Monica, CA) were used:

- Cl‐PDCD1 (Cat No. 488499) for PD‐1;
- Cl‐CD274 (Cat No. 488469) for PD‐L1;
- Cl‐CTLA4 (Cat No. 488479) for CTLA‐4.

Briefly, 4‐μm FFPE tissue serial sections were deparaffinised and permeabilised with 2.5 VS Protease (ACD Inc.) at 37°C for 16 min. The probes were incubated at 42°C for 2 h. The final deposit was detected as a red punctate precipitate using the RED mRNA detection kit (Ventana Medical System Inc.). For each sample, 2 serial sections were stained using CI‐PPIB (peptidylprolyl isomerase B [cyclophilin B]) and dapB ( *Bacillus subtilis* dihydrodipicolinate reductase [dapB]) probes. PPIB was used as an endogenous control to evaluate RNA integrity, and the bacterial gene dapB served as a negative control to assess nonspecific staining. For highly pigmented tumours, slides were bleached with a 3% H_2_O_2_ solution in PBS at 65°C for 30 min.

RNAscope expression of PD‐L1, PD‐1 and CTLA‐4 in tumour cells (pigmented, or nonpigmented atypical cells with vesicular nuclei and prominent nucleoli) was assessed and semi‐quantified into 5 scores (0–4) based on the ACD scoring system [25]. In cases where positivity was not uniformly distributed, the overall average score was estimated.

Moreover, automated quantitative analysis of PD‐L1, PD‐1 and CTLA‐4 RNAscope expression on the whole tumour area (including tumour cells, stromal areas and TME immune cells, with exclusion of areas of necrosis, pigmentation or close to ulceration) was performed using QuPath (version 0.4.0) [26]. Slides were digitised with the scanner Aperio LV1 (Leica, Wetzlar, Germany) using a resolution of 40×. Positive RNAscope signals were detected using “cell detection” and “subcellular detection” functions. The ratio of estimated dots/number of cells was obtained.

### 2.4 Dual ISH/IHC

To localise the expression of immune checkpoints, dual ISH/IHC was performed on 5 representative cases with the Discovery ULTRA system (Ventana Medical System Inc.), using the PD‐1, PD‐L1, CTLA‐4 RNAscope probes and the primary antibodies for Melanoma Triple Cocktail (Ventana Medical System Inc., code 06527787001), CD3 (Dako), CD20 (Thermo Fisher Scientific) and Iba1 (FUJIFILM Wako, Pure Chemical Corporation, code 019‐19741) for melanocytic neoplastic cells, T lymphocytes, B lymphocytes and tumour‐associated macrophages (TAMs), respectively [27].

Dual ISH/IHC integrated approach was performed for the Melanoma Triple Cocktail, CD3 and CD20 using the VS RNA‐Protein Co‐detection Ancillary Kit (ACD Inc.). Briefly, antigen retrieval and incubation with the primary antibodies were followed by fixation of tissue slides with 10% neutral buffered formalin, permeabilisation with VS Co‐Detection Protease, incubation with RNAscope probes and amplification of ISH signal with RED mRNA detection kit. IHC signal was detected using the Discovery Green HRP chromogen (Ventana Medical System Inc.). Dual ISH/IHC sequential approach was performed for Iba1. After antigen retrieval and permeabilisation, RNAscope ISH and IHC were performed sequentially using the RED mRNA detection kit and the Discovery Green HRP chromogen, respectively. Details about the primary antibodies, antigen retrieval, detection systems and positive controls are listed in Table 1.

**TABLE 1:** List of antibodies and immunohistochemistry (IHC) protocols used in dual RNAscope in situ hybridization (ISH)/IHC combined with PD‐1, PD‐L1, and CTLA‐4 probes.

Antibody	Clone	Antigen retrieval	Antibody incubation	Detection system	Positive controls^a^
Melanoma Triple Cocktail	Monoclonal anti‐melanosome (HMB45), anti‐MART1/melan A (A103), anti‐tyrosinase (T311)	CC1 ^b^ 97°C 56 min	RTU 60 min RT	Discovery OmniMap anti‐mouse HRP^b^	Oral melanoma
CD3	Polyclonal	CC1 97°C 48 min	1:50 48 min RT	Discovery OmniMap anti‐rabbit HRP^b^	Lymph node
CD20	Polyclonal	CC1 97°C 32 min	1:100 60 min RT	Discovery OmniMap anti‐rabbit HRP	Lymph node
Iba1	Polyclonal	CC1 97°C 24 min	1:1000 32 min 36°C	Discovery anti‐rabbit HQ^b^, Anti‐HQ HRP^b^	Cutaneous histiocytoma

Abbreviations: CC1 = cell conditioning solution 1, pH 8.4; RT = room temperature; RTU = ready to use.

^a^ Canine tissues.

^b^ Ventana Medical System Inc., Roche (Tucson, AZ, USA).

### 2.5 Statistical Analysis

The RNAscope ACD scores were analysed in pairwise comparisons using the non‐parametric Sign test.

Descriptive statistics for quantitative variables were expressed as median and interquartile range (IQR). For the following statistical analysis, cases were stratified as follows: PD‐L1 ACD score = 1 versus score ≥ 2; PD‐1 and CTLA‐4 ACD score = 0 versus score ≥ 1; TILs grades 0–1 versus 2–3. The Wilcoxon's rank‐sum test was used to evaluate differences in the distribution of quantitative parameters with respect to ACD RNAscope scores and TILs grades.

For qualitative variables, the Fisher's exact test was used to evaluate significant associations with the RNAscope scores. The Kaplan–Meier method was used to draw the cumulative survival curves for the RNAscope scores, and TILs grades, considering the OS and DFI. The dogs alive at the end of the follow‐up or those dead for other causes were censored using the time from diagnosis to their latest follow‐up. The Wilcoxon test was adopted to compare survival curves among groups. The correlations between the expression of PD‐L1 versus PD‐1, PD‐L1 versus CTLA‐4 and PD‐1 versus CTLA‐4 on the whole tumour area were evaluated by the Spearman's rank correlation coefficient. Statistical significance was set at *p* value < 0.05. All statistical analyses were carried out using STATA version 17.0.

## 3 Results

### 3.1 Cases

Twenty‐four canine OMs were included in the study. Data regarding signalment and clinical features are summarised in Table S1. The age at the time of diagnosis ranged from 7.5 to 16 years with a mean age of 11.1 years. Ten dogs (42%) were females and 14 (58%) were males. Eight dogs (33%) were mixed breed, and the most represented breed was Golden Retriever (*n* = 4, 17%). Melanomas developed in the mandible (*n* = 13, 54%), maxilla (*n* = 3, 12.5%), lip (*n* = 2, 8%) and cheek (*n* = 2, 8%). The specific tumour localization was not recorded for 4 cases. At the time of diagnosis, melanomas were staged as stage I (*n* = 1, 4%), stage II (*n* = 5, 21%), stage III (*n* = 16, 67%) and stage IV (*n* = 2, 8%) according to the World Health Organisation guidelines [28, 29]. Information about surgical margins were available for 23/24 cases, being clean in 18 cases (75%) and infiltrated in 5 cases (21%).

In addition to surgery, 8 out of 24 dogs (33%) received immunotherapy with an anti‐chondroitin sulphate proteoglycan‐4 (CSPG4) DNA‐based vaccine [18] and metronomic chemotherapy. Three dogs (12.5%) were treated with immunotherapy alone, 3 (12.5%) with chemotherapy alone, 2 (8%) with locoregional electrochemotherapy and 5 (21%) received no further treatment after surgery.

Follow‐up data were available for 23 dogs. One dog was euthanized at the time of diagnosis and was excluded from the survival analysis. Twenty out of 23 (87%) dogs experienced recurrence or metastasis with a median DFI of 116 days (IQR: 60–256). Seventeen of 23 dogs (74%) died because of the tumour; the median OS was 335 days (IQR: 96–598).

### 3.2 Histological Parameters and TILs Assessment

Based on cell morphology, tumours were classified as mixed (*n* = 16, 66%), spindle cell (*n* = 5, 21%) and epithelioid (*n* = 3, 12.5%) melanomas. Tumour pigmentation was observed in 16 cases (67%), with percentages of pigmented cells ranging from 2% to 100%. Necrosis was present in 12 cases (50%), occupying 2%–30% of tumour section. The percentage of cells with nuclear atypia ranged from 25% to 100%. Lymphovascular invasion was observed in 2 cases.

TILs were graded as grade 0 in 3 cases (12%), grade 1 in 6 cases (25%), grade 2 in 10 cases (42%) and grade 3 in 5 cases (21%). TILs grade was not associated with DFI and OS (Figures S2 and S3).

### 3.3 RNAscope ISH

**FIGURE 1:**
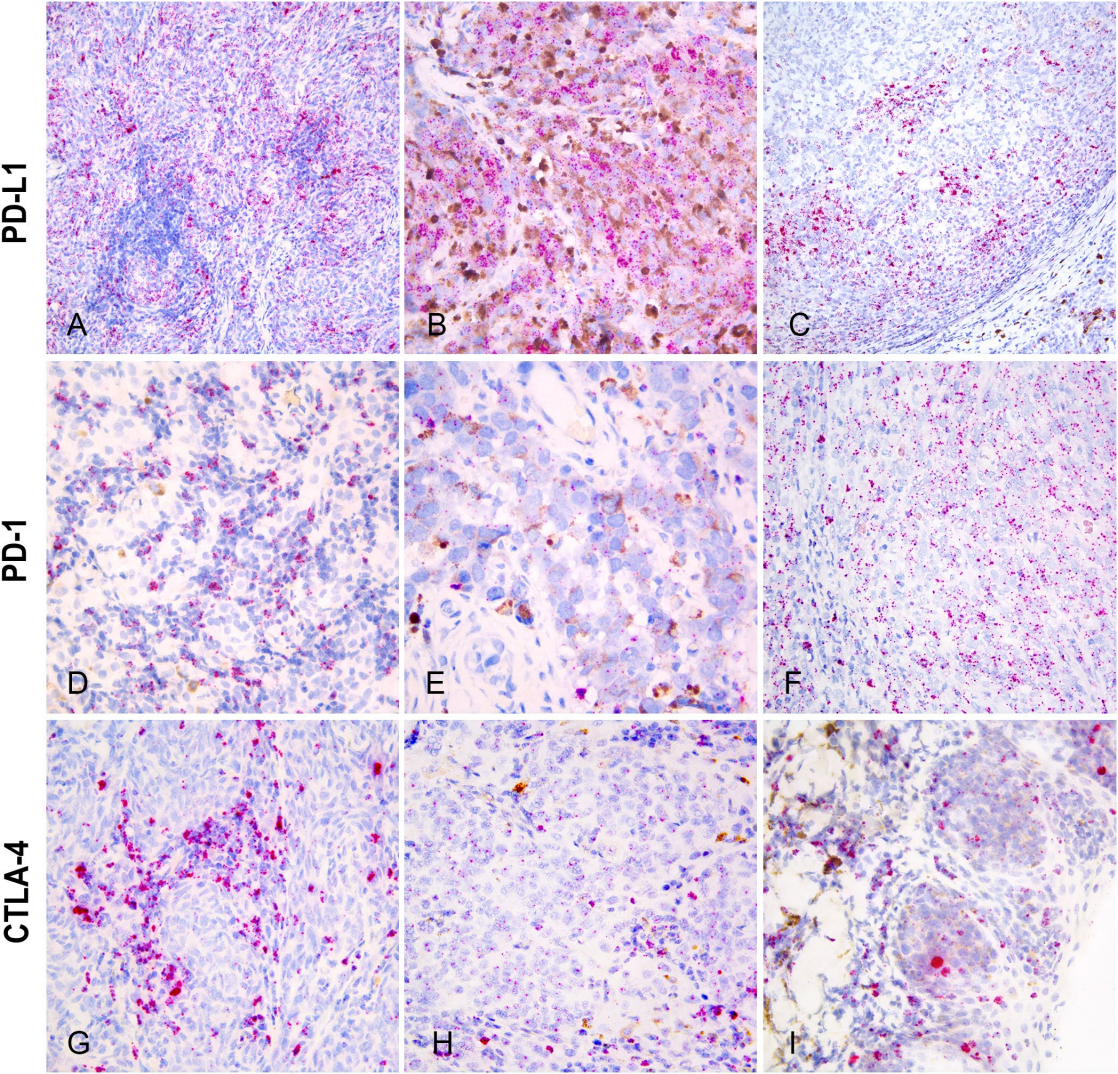
PD‐L1, PD‐1 and CTLA‐4 mRNA expression by RNAscope in situ hybridization (ISH) in canine melanoma tumour microenvironment (RED mRNA detection kit, Haematoxylin II [Ventana Medical System Inc.]). (A) High PD‐L1 expression (score 3) in proximity of tumour‐infiltrating lymphocytes (TILs) aggregates (20× magnification). (B) High PD‐L1 expression (score 3) in pigmented melanoma cells (40×). (C) PD‐L1 increased expression near host‐tumour interface (20×). (D) PD‐1 multifocal expression in large clusters within cells with scant cytoplasm and round hyperchromatic nuclei, interpreted as small lymphocytes (40×). (E) Low PD‐1 expression (score 1) within poorly pigmented melanoma cells (63×). (F) Moderate PD‐1 expression (score 2) within large cells with vesicular nuclei interpreted as amelanotic tumour cells (40×). (G) High CTLA‐4 expression in large clusters within cells with scant cytoplasm and round hyperchromatic nuclei, interpreted as small lymphocytes. Neoplastic nests exhibit low CTLA‐4 expression (score 1), (40×). (H) Low CTLA‐4 expression (score 1) within large cells with vesicular nuclei interpreted as amelanotic tumour cells (40×). (I) Low CTLA‐4 expression (score 1) within poorly pigmented neoplastic nests (40×).

RNAscope positive signals appeared as cytoplasmic red punctate dots or clusters (Figure 1). Expression of PD‐L1, PD‐1 and CTLA‐4 mRNA was observed in pigmented and/or non‐pigmented atypical cells with vesicular nuclei and prominent nucleoli interpreted as tumour cells, as well as in non‐tumour cells, mostly characterised by scant cytoplasm, interpreted as small lymphocytes (Figure 1A,D,G).

**FIGURE 2:**
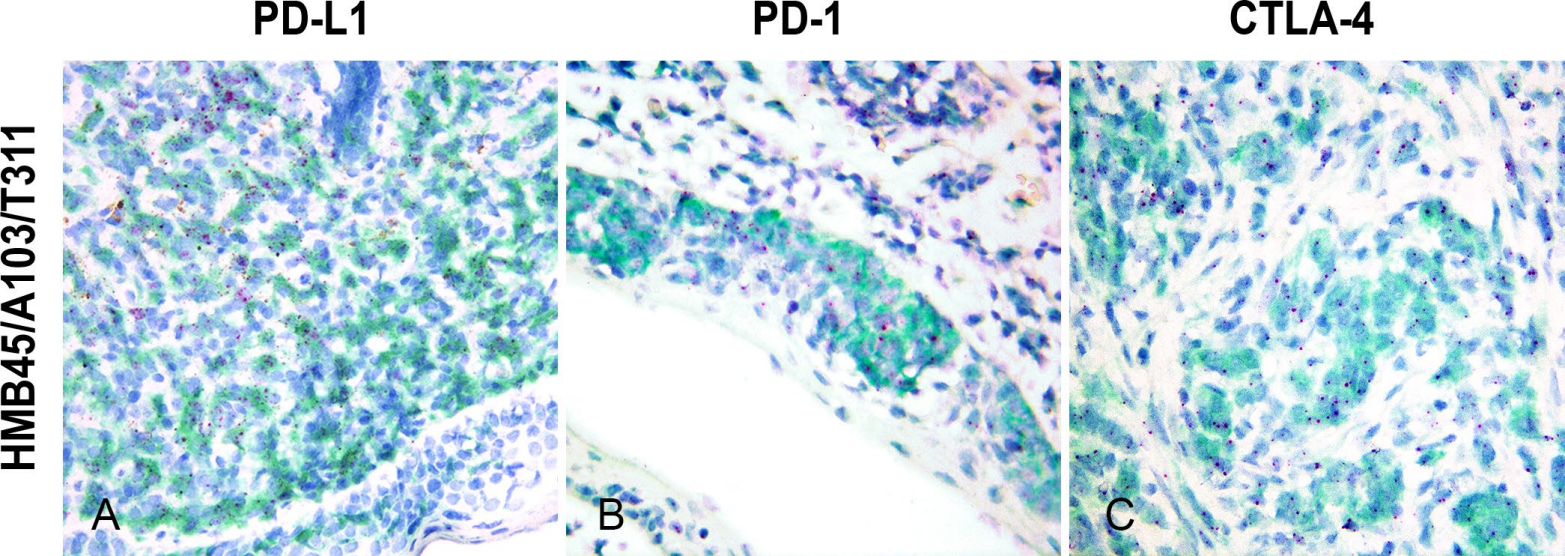
PD‐L1, PD‐1 and CTLA‐4 mRNA expression localised within melanoma cells detected by dual in situ hybridization (RED mRNA detection kit)/immunohistochemistry for Melanoma Triple Cocktail (Discovery Green HRP chromogen, Haematoxylin II). (A) Moderate PD‐L1 expression (score 2) within melanoma cells infiltrating the lamina propria (40× magnification). (B) Low PD‐1 expression (score 1) within melanoma intraepithelial nests (63×). (C) Low CTLA‐4 expression (score 1) within melanoma cells (63×).

PD‐L1 expression was detected in tumour cells in all cases (Figures 1A–C and 2A). PD‐L1 score 1 was observed in 12 out of 24 (50%) cases, score 2 (Figure 1C) in 10/24 (42%) cases and score 3 (Figure 1A,B) in 2/24 (8%) cases. The distribution of PD‐L1 expression in tumour cells was multifocal in 17/24 (71%) and diffuse in 7/24 (29%) cases. Higher PD‐L1 scores were observed in cases with multifocal rather than diffuse expression (*p* = 0.002). PD‐L1 expression was usually higher in proximity of TILs aggregates (Figure 1A) and at the host‐tumour interface (Figure 1C).

PD‐1 expression was detected in neoplastic cells in 13 out of 24 (54%) cases (Figures 1E,F and 2B), with 12 cases (50%) scored as score 1 (Figure 1E) and one case (4%) as score 2 (Figure 1F). The staining distribution was multifocal in 10/13 (76.9%) cases and diffuse in 3/13 (30.8%) cases. In 11 tumours (46%), no or minimal PD‐1 expression was detected in neoplastic cells (score 0).

CTLA‐4 expression was detected in tumour cells in 18 out of 24 (75%) cases, all as score 1 (Figure 1G–I). The distribution of CTLA‐4 expression was diffuse in 10/18 (56%) and multifocal in 8/24 (44%) cases. In 6/24 (25%) cases, CTLA‐4 signal was minimal or absent (score 0).

PD‐L1 semi‐quantitative scores in neoplastic cells were higher compared to PD‐1 and CTLA‐4 (*p <* 0.001 for both comparisons).

PD‐1 expression score in neoplastic cells was associated with a higher mitotic count (*p* = 0.006) (Table 2). The expression scores of PD‐L1, PD‐1 and CTLA‐4 in neoplastic cells were not correlated with Ki67 index, percentage of nuclear atypia, CD3+/CD20+ cell counts (Table 2), TILs grade, DFI, or OS (Figures S2 and S3).

**TABLE 2:** Distribution of quantitative parameters relative to mitotic count, Ki67 index, nuclear atypia and counts of CD3+/CD20+ positive tumour infiltrating lymphocytes with respect to PD‐L1, PD‐1 and CTLA‐4 ACD RNAscope semi‐quantitative scores in neoplastic cells (Wilcoxon's rank‐sum test).

		PD‐L1			PD‐1			CTLA‐4		
Parameter		ACD score = 1 (*n* = 12)	ACD score ≥ 2 (*n* = 12)	*p*	ACD score = 0 (*n* = 11)	ACD score ≥ 1 (*n* = 13)	*p*	ACD score = 0 (*n* = 6)	ACD score = 1 (*n* = 18)	*p*
Mitotic count	Median	26.50	34.50	ns	21.00	42.00	0.006	30.50	28.50	ns
	IQR	41.50	34.50		21.00	34.00		82.00	26.00	
Ki67 index	Median	54.70	58.10	ns	46.20	60.80	ns	34.30	59.90	ns
	IQR	48.10	27.90		67.20	27.60		59.60	45.60	
Nuclear atypia	Median	70.00	60.00	ns	60.00	70.00	ns	62.50	67.50	ns
	IQR	27.50	32.50		40.00	30.00		35.00	30.00	
CD3+ cells	Median	21.30	38.10	ns	21.40	21.20	ns	8.80	22.60	ns
	IQR	22.50	64.90		29.80	46.80		58.20	26.00	
CD20+ cells	Median	3.20	9.50	ns	7.40	4.40	ns	6.40	5.90	ns
	IQR	12.50	35.70		12.40	24.00		25.00	21.40	

*Note: p* value > 0.05.

Abbreviations: IQR, interquartile range; ns, not significant.

Image analysis was performed in 19 cases for PD‐L1, 16 for PD‐1 and 17 for CTLA‐4. Remaining cases were excluded due to the sub‐optimal scanned slides quality. A significant positive correlation between PD‐1 and CTLA‐4 expression on the whole tumour area was observed (Spearman's rank correlation coefficient = 0.56, *p* = 0.025). Whole tumour PD‐L1 expression was significantly correlated to PD‐L1 scoring in neoplastic cells (*p* = 0.003) (Table 3). Whole tumour PD‐1 expression was significantly correlated to the TILs grade (*p* = 0.003) (Table 4).

**TABLE 3:** Distribution of quantitative whole tumour PD‐L1, PD‐1 and CTLA‐4 in situ RNAscope expression (estimated dot/cell) with respect to relative ACD semi‐quantitative scores in neoplastic cells (Wilcoxon's rank‐sum test).

		PD‐L1			PD‐1			CTLA‐4		
Parameter		ACD score = 1 (*n* = 9)	ACD score ≥ 2 (*n* = 10)	*p*	ACD score = 0 (*n* = 5)	ACD score ≥ 1 (*n* = 12)	*p*	ACD score = 0 (*n* = 1)	ACD score = 1 (*n* = 17)	*p*
Estimated dot/cell	Median	0.35	1.37	0.003	0.17	0.20	ns	2.50	0.49	na
	IQR	0.28	0.64		0.03	0.23			0.59	

*Note: p* value > 0.05.

Abbreviations: IQR, interquartile range; na, not available; ns, not significant.

**TABLE 4:** Distributions of quantitative whole tumour PD‐L1, PD‐1 and CTLA‐4 in situ RNAscope expression with respect to the grade of tumour infiltrating lymphocytes (TILs) (Wilcoxon's rank‐sum test).

		PD‐L1			PD‐1			CTLA‐4		
Parameter		TILs 0–1 (*n* = 6)	TILs 2–3 (*n* = 13)	*p*	TILs 0–1 (*n* = 6)	TILs 2–3 (*n* = 11)	*p*	TILs 0–1 (*n* = 6)	TILs 2–3 (*n* = 12)	*p*
Estimated dot/cell	median	0.86	0.67	ns	0.12	0.31	0.003	0.54	0.43	ns
	IQR	1.48	0.81		0.09	0.28		0.48	1.49	

*Note: p* value 0.05.

Abbreviations: IQR, interquartile range; ns, not significant.

### 3.4 Dual ISH/IHC

Dual staining with Melanoma Triple Cocktail enabled confirmation of PD‐L1, PD‐1 and CTLA‐4 expression by neoplastic melanocytic cells (Figure 2).

**FIGURE 3:**
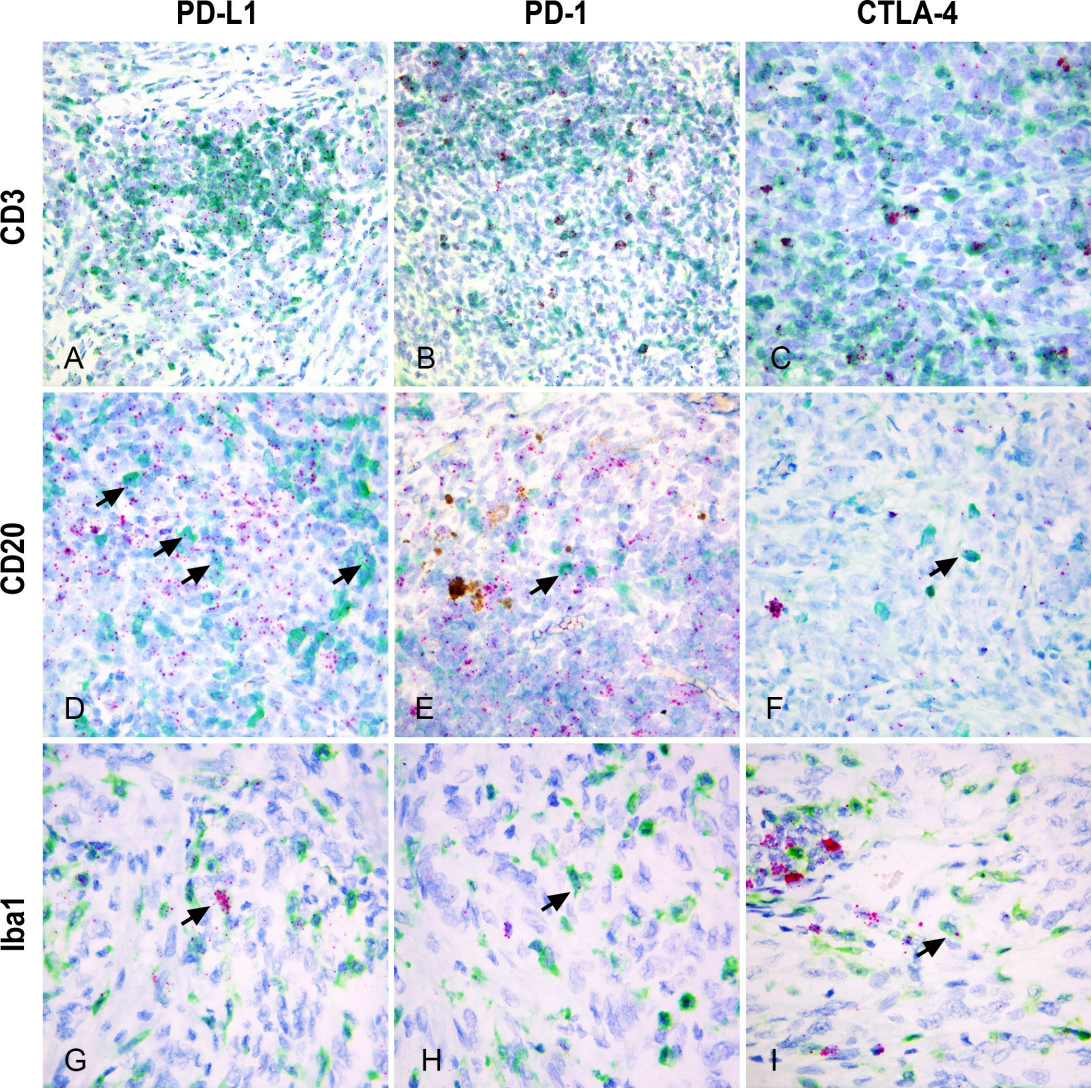
PD‐L1, PD‐1 and CTLA‐4 mRNA expression localised within immune cells of the tumour microenvironment detected by dual in situ hybridization (RED mRNA detection kit)/immunohistochemistry for CD3 (T lymphocytes), CD20 (B lymphocytes), Iba1 (Tumour‐associated macrophages, TAMs), (Discovery Green HRP chromogen, Haematoxylin II). (A) PD‐L1 moderate expression within CD3+ tumour‐infiltrating lymphocytes (TILs) aggregates (40× magnification). (B) (40×), (C) (63×). Scattered CD3+ small lymphocytes express high quantities of PD‐1 and CTLA‐4, respectively, as large clusters partially obscuring the nucleus. (D–F) Rare CD20+ lymphocytes express low quantities of PD‐L1, PD‐1 and CTLA‐4 (arrows), respectively, as single dots, while the majority of PD‐L1‐, PD‐1‐ and CTLA‐4‐expressing lymphocytes are CD20‐ (63×). (G) Iba1+ TAMs expressing low to high (arrow) levels of PD‐L1 (63×). (H, I) Rare Iba1+ macrophages express low quantities of PD‐1 and CTLA‐4 (arrows), respectively, as single dots (63×).

CD3+ T lymphocytes frequently exhibited mRNA expression of PD‐L1, PD‐1 and CTLA‐4 (Figure 3A–C), particularly for PD‐1 and CTLA‐4, which displayed large clusters obscuring the nucleus in a subset of T cells (Figure 3B,C). The expression of PD‐L1, PD‐1 and CTLA‐4 was sporadically observed in CD20+ B lymphocytes, as individual dots (Figure 3D–F). In Iba1+ macrophages, PD‐L1 was commonly expressed, often as multiple dots per cell or in large clusters (Figure 3G). Conversely, PD‐1 and CTLA‐4 expression was occasionally detected in Iba1+ cells (Figure 3H,I).

## 4 Discussion

Immune checkpoint pathways are exploited by cancer cells to suppress anti‐tumour immunity. PD‐1/PD‐L1 and CTLA‐4 are clinically important immune checkpoints in human melanoma, as the introduction of ICI treatment has significantly improved the outcome for patients with advanced disease.

Data on immune checkpoint expression in canine OM tissue are essential for understanding immune evasion mechanisms and supporting the use of targeted immunotherapies, also in view of its potential role as a clinical model for human mucosal melanoma [4].

In this study, we demonstrated the in situ mRNA expression of PD‐L1, PD‐1 and CTLA‐4 in both tumour cells and immune cells of the TME in canine OM.

PD‐L1 was the most highly expressed immune checkpoint in neoplastic cells, highlighting its role in promoting immune tolerance. In our study, 100% of canine OMs exhibited mRNA PD‐L1 in situ expression, consistent with previous studies reporting PD‐L1 positivity in 90%–100% of cases using IHC or IF [19, 20, 21]. PD‐L1 immunohistochemical expression in human melanoma is less prevalent, varying across studies with different cut‐off values and reagents [9, 30, 31]. However, to definitively compare the discrepancies between human and canine tumours, it is imperative to employ methods with comparable sensitivity and scoring systems.

In this study, PD‐L1 expression in the whole tumour area, comprising tumour cells, the stroma and immune cells, was correlated with its expression in melanoma cells, suggesting that tumour cells are key contributors to the activation of the PD‐1/PD‐L1 immunoinhibitory axis. PD‐L1 is typically expressed by antigen‐presenting cells, such as macrophages and dendritic cells, and in some activated T and B cells [32]. In tumour cells, PD‐L1 expression can be induced by signals from immune cells in the TME [5]. Interferon‐gamma (IFN‐γ), produced by T or NK lymphocytes, is a major cytokine that induces PD‐L1 expression through activating the JAK/STAT signalling pathway, as demonstrated in canine melanoma cells [33, 34, 35]. TNF‐α also induced PD‐L1 overexpression through NF‐κB pathway in one canine melanoma cell line [34]. Alternatively, PD‐L1 expression can be associated to constitutive oncogenic pathways of tumour cells [5]. Most cases in this study exhibited geographically heterogeneous PD‐L1 expression, with higher intensity observed near stromal areas and lymphocyte infiltrates, similar to what observed with PD‐L1 IHC in human melanomas [36]. Moreover, multifocal PD‐L1 expression correlated with higher PD‐L1 scores, supporting that PD‐L1 expression in canine OM may be primarily induced by immune activity at the host‐tumour interface.

In humans, PD‐L1 expression in melanocytic cells is associated with the presence of TILs and generally reflects an immunoactive TME [37]. Classification of tumours on the basis of their PD‐L1 immunohistochemical status and presence or absence of TILs has been proposed [38], with the PD‐L1+/TILs+ phenotype being the most responsive to checkpoint blockade [37]. In our study, although increased PD‐L1 expression was observed near TILs aggregates, no correlation was found between TILs grades and PD‐L1 scores. This may be related to the small sample size. A previous study on canine melanomas showed a correlation between higher global PD‐L1 gene expression and increased CD3+ cells [39].

PD‐L1 mRNA expression was observed also in melanoma‐infiltrating immune cells, as previously demonstrated by IHC and IF [20, 21], although its localization within specific cell types has not been investigated. RNAscope ISH/IHC double staining revealed that PD‐L1 is predominantly expressed by Iba1+ TAMs, to a lesser extent in CD3+ T cells, and rarely by CD20+ B cells, highlighting the role of diverse immune cells in mediating local immunosuppression and potentially influencing the response to immunotherapy.

The overexpression of PD‐L1 by tumour cells is a primary mechanism through which they evade elimination by PD‐1‐expressing, tumour‐specific T lymphocytes. PD‐1 is expressed on non‐naive T cells, including activated memory and senescent T cells, as well as on activated NK cells, dendritic cells, monocytes and mature B lymphocytes [6]. In our study, PD‐1 in situ gene expression was commonly observed in TILs, particularly CD3+ T cells and less so in Iba1+ TAMs and CD20+ B cells. This finding is consistent with previous results revealing PD‐1 expression by flow cytometry on both CD8+ and CD4+ TILs from canine OM [19], suggesting the presence of exhausted T cells within the TME that could potentially respond to ICIs. Moreover, image analysis of whole tumour sections revealed a significant association between PD‐1 expression and TILs grades, aligning with earlier studies demonstrating correlation between PD‐1 gene expression and CD3+ cells densities [4, 39].

In our study, approximately half of cases exhibited PD‐1 gene expression in neoplastic melanocytic cells, primarily with low levels and multifocal distribution. PD‐1 expression has also been demonstrated in cell subpopulations of human melanoma and canine diffuse large B‐cell lymphoma [25, 40]. This finding is critically important, since the growth‐suppressive effects of anti‐PD‐1 therapy might also result from the direct inhibition of this protein on neoplastic cells rather than on immune cells [40]. Interestingly, the observed association between PD‐1 expression level in tumour cells and higher mitotic counts suggests a potential role of PD‐1 in promoting tumour growth, similar to what has been postulated in human melanoma where PD‐1 mediates mTOR pathway activity [40]. This warrants further investigation to clarify whether PD‐1 expression may contribute to tumourigenesis, independently of its immunosuppressive function.

The demonstration of PD‐1 and PD‐L1 expression in the TME of canine OM supports the use of ICIs targeting the PD‐1/PD‐L1 axis. Initial attempts of immunotherapy with ICIs have been made in dogs with OM, though further studies are required to assess their clinical efficacy [12, 13, 19, 21, 41, 42]. Treatment with the anti‐PD‐1 antibody gilvetmab, recently conditionally approved for canine melanoma, resulted in a 60% clinical benefit rate and a 20% objective response rate [41]. A different trial using a distinct anti‐PD‐1 antibody reported a 26% objective partial response in dogs with stage IV OM [12]. A pilot study of anti‐PD‐L1 therapy showed improved OS in dogs with OM and lung metastasis, with enhanced clinical response when combined with hypofractionated radiation therapy [21, 43]. Despite these preliminary results, the association between immune checkpoints expression in tumour tissue and clinical outcomes remains underexplored. In one of the cited trials, the tumour proportion score (TPS), a scoring method adapted from human criteria, was used to quantify PD‐L1 IHC expression in OM tissue. While most dogs had TPS ≥ 50%, among five dogs that responded to treatment, one had TPS of < 1%, suggesting that PD‐L1 IHC alone might not be a sufficient biomarker of therapy response [21].

CTLA‐4 suppresses T cell proliferation at the initial stage of naive T‐cell activation, primarily in lymph nodes [44]. It is a member of the family of immunoglobulin‐related receptors, expressed on both activated conventional lymphocytes and Tregs [44]. In our study, CTLA‐4 mRNA in situ expression was detected on T cells, consistent with previous studies on canine OM demonstrating CTLA‐4 immunohistochemical expression in TILs and the correlation of increased CTLA‐4 gene expression with higher T cell infiltration [4, 22]. A proportion of CD3+ cells exhibited elevated expression, possibly indicating a state of exhaustion [7]. Occasional positivity in CD20+ TILs and Iba1+ TAMs was also detected. Indeed, CTLA‐4 is not restricted to the lymphoid cell lineage, as it has been demonstrated in B and myeloid cells, underscoring the intricate interplay of immune suppressive mechanisms among immune cells [45].

Interestingly, CTLA‐4 mRNA was detected in melanoma cells of 75% tested canine OMs, with low expression levels. While CTLA‐4 expression in canine tumours has not been previously reported, several studies have shown its induction in neoplastic cells of human solid tumours, including melanoma [46, 47]. Although the role of CTLA‐4 upregulation in tumour cells remains unclear, the level of CTLA‐4 expression in melanoma cells has been associated with clinical response to the CTLA‐4 inhibitor ipilimumab, supporting its possible role as a predictive biomarker [47].

In our study, the level of PD‐L1, PD‐1, CTLA‐4 mRNA expression in neoplastic cells was not associated with DFI or OS, suggesting that these markers lack prognostic relevance in canine OM. This finding may reflect the complex multifactorial nature of immune evasion, influenced by both intrinsic tumour biology and TME. However, limitations of our cohort, such as the small sample size, the variability in clinical characteristics and treatment regimens, may have obscured potential correlations. The prognostic significance of immune checkpoint expression in melanoma cells remains controversial in humans. Recent meta‐analyses support that PD‐L1 expression is not predictive of inferior prognosis, as previously suggested [48].

In conclusion, our study demonstrated mRNA expression of PD‐L1, PD‐1 and CTLA‐4 by both neoplastic and immune cells in canine OMs, supporting their role in immunosuppression and in promoting tumour progression. PD‐L1 testing by IHC is the most widely used predictive biomarker for selecting human patients eligible for immunotherapy targeting PD‐1/PD‐L1 [10]. In veterinary medicine, technical aspects, such as pre‐analytical bias, the use of commercial antibodies designed for human tissues, even though with cross‐reactivity for canine tissues [20], and the lack of reference scoring methods, provide discordant and less reliable results. While the validation of anti‐canine PD‐1/PD‐L1 antibodies is under study [17, 49], RNAscope ISH represents a valuable alternative for analysing the spatial distribution of these molecules within tissue sections, providing a deeper understanding of immunosuppressive interactions between tumour cells and the TME. Also, the co‐localization investigated by analysing multiple markers simultaneously via dual ISH/IHC gives insight into the cellular localization and functional relationships between molecules. Further studies are warranted to determine whether ISH may guide the selection of the patients that benefit from ICI therapy.

## Ethics Statement

The authors confirm that the research presented in this manuscript adheres to a high standard of veterinary care for client‐owned animals and involves informed consent from clients.

## Conflicts of Interest

The authors declare no conflicts of interest.

## Supporting information


**Data S1.** Supporting Information.

## Data Availability

The data that support the findings of this study are available from the corresponding author upon reasonable request.

## References

[1] R. C. Smedley , K. Sebastian , and M. Kiupel , “Diagnosis and Prognosis of Canine Melanocytic Neoplasms,” Veterinary Sciences 9, no. 4 (2022): 175. 35448673 10.3390/vetsci9040175 PMC9030435

[2] V. B. Stevenson , S. Klahn , T. LeRoith , and W. R. Huckle , “Canine Melanoma: A Review of Diagnostics and Comparative Mechanisms of Disease and Immunotolerance in the Era of the Immunotherapies,” Frontiers in Veterinary Science 9 (2023): 1046636. 36686160 10.3389/fvets.2022.1046636 PMC9853198

[3] G. Che , B. Huang , Z. Xie , et al., “Trends in Incidence and Survival in Patients With Melanoma, 1974–2013,” American Journal of Cancer Research 9, no. 7 (2019): 1396–1414. 31392077 PMC6682720

[4] K. E. Cronise , J. Coy , S. Dow , M. L. Hauck , and D. P. Regan , “Immunohistochemical and Transcriptomic Characterization of T and Myeloid Cell Infiltrates in Canine Malignant Melanoma,” Veterinary and Comparative Oncology 22, no. 3 (2024): 377–387. 38752589 10.1111/vco.12981 PMC11323233

[5] S. L. Topalian , C. G. Drake , and D. M. Pardoll , “Immune Checkpoint Blockade: A Common Denominator Approach to Cancer Therapy,” Cancer Cell 27, no. 4 (2015): 450–461. 25858804 10.1016/j.ccell.2015.03.001 PMC4400238

[6] M. E. Keir , M. J. Butte , G. J. Freeman , and A. H. Sharpe , “PD‐1 and Its Ligands in Tolerance and Immunity,” Annual Review of Immunology 26 (2008): 677–704. 10.1146/annurev.immunol.26.021607.090331 PMC10637733 18173375

[7] C. Tay , A. Tanaka , and S. Sakaguchi , “Tumor‐Infiltrating Regulatory T Cells as Targets of Cancer Immunotherapy,” Cancer Cell 41, no. 3 (2023): 450–465. 36917950 10.1016/j.ccell.2023.02.014

[8] J. D. Wolchok , V. Chiarion‐Sileni , R. Gonzalez , et al., “Long‐Term Outcomes With Nivolumab Plus Ipilimumab or Nivolumab Alone Versus Ipilimumab in Patients With Advanced Melanoma,” Journal of Clinical Oncology 40, no. 2 (2022): 127–137. 34818112 10.1200/JCO.21.02229 PMC8718224

[9] S. Yentz and C. D. Lao , “Immunotherapy for Mucosal Melanoma,” Annals of Translational Medicine 7 (2019): S118. 31576325 10.21037/atm.2019.05.62 PMC6685869

[10] S. Vranic and Z. Gatalica , “PD‐L1 Testing by Immunohistochemistry in Immuno‐Oncology,” Biomolecules and Biomedicine 23, no. 1 (2023): 15–25. 35964287 10.17305/bjbms.2022.7953 PMC9901897

[11] N. Maekawa , S. Konnai , S. Takagi , et al., “A Canine Chimeric Monoclonal Antibody Targeting PD‐L1 and Its Clinical Efficacy in Canine Oral Malignant Melanoma or Undifferentiated Sarcoma,” Scientific Reports 7, no. 1 (2017): 8951. 28827658 10.1038/s41598-017-09444-2 PMC5567082

[12] M. Igase , Y. Nemoto , K. Itamoto , et al., “A Pilot Clinical Study of the Therapeutic Antibody Against Canine PD‐1 for Advanced Spontaneous Cancers in Dogs,” Scientific Reports 10, no. 1 (2020): 1–16. 33110170 10.1038/s41598-020-75533-4 PMC7591904

[13] M. Igase , S. Inanaga , K. Tani , et al., “Long‐Term Survival of Dogs With Stage 4 Oral Malignant Melanoma Treated With Anti‐Canine PD‐1 Therapeutic Antibody: A Follow‐Up Case Report,” Veterinary and Comparative Oncology 20, no. 4 (2022): 901–905. 35535636 10.1111/vco.12829

[14] J. Marable , D. Ruiz , A. K. Jaiswal , et al., “Nanobody‐Based CTLA4 Inhibitors for Immune Checkpoint Blockade Therapy of Canine Cancer Patients,” Scientific Reports 11, no. 1 (2021): 20763. 34675296 10.1038/s41598-021-00325-3 PMC8531395

[15] N. J. Mason , N. Chester , A. Xiong , et al., “Development of a Fully Canine Anti‐Canine CTLA4 Monoclonal Antibody for Comparative Translational Research in Dogs With Spontaneous Tumors,” MAbs 13, no. 1 (2021): 2004638. 34856888 10.1080/19420862.2021.2004638 PMC8726733

[16] S. Yoshimoto , N. Chester , A. Xiong , et al., “Development and Pharmacokinetic Assessment of a Fully Canine Anti‐PD‐1 Monoclonal Antibody for Comparative Translational Research in Dogs With Spontaneous Tumors,” MAbs 15, no. 1 (2023): 2287250. 38047502 10.1080/19420862.2023.2287250 PMC10793675

[17] S. Sirivisoot , C. Boonkrai , T. Wongtangprasert , et al., “Development and Characterization of Mouse Anti‐Canine PD‐L1 Monoclonal Antibodies and Their Expression in Canine Tumors by Immunohistochemistry In Vitro,” Veterinary Quarterly 43, no. 1 (2023): 1–9. 10.1080/01652176.2023.2240380 PMC10388796 37477617

[18] L. A. Piras , F. Riccardo , S. Iussich , et al., “Prolongation of Survival of Dogs With Oral Malignant Melanoma Treated by en Bloc Surgical Resection and Adjuvant CSPG4‐Antigen Electrovaccination,” Veterinary and Comparative Oncology 15, no. 3 (2017): 996–1013. 27146852 10.1111/vco.12239 PMC8668196

[19] N. Maekawa , S. Konnai , T. Okagawa , et al., “Immunohistochemical Analysis of PD‐L1 Expression in Canine Malignant Cancers and PD‐1 Expression on Lymphocytes in Canine Oral Melanoma,” PLoS One 11, no. 6 (2016): 1–13. 10.1371/journal.pone.0157176 PMC4898770 27276060

[20] L. V. Muscatello , F. Gobbo , G. Avallone , et al., “PDL1 Immunohistochemistry in Canine Neoplasms: Validation of Commercial Antibodies, Standardization of Evaluation, and Scoring Systems,” Veterinary Pathology 61, no. 3 (2024): 393–401. 37920996 10.1177/03009858231209410

[21] N. Maekawa , S. Konnai , M. Nishimura , et al., “PD‐L1 Immunohistochemistry for Canine Cancers and Clinical Benefit of Anti‐PD‐L1 Antibody in Dogs With Pulmonary Metastatic Oral Malignant Melanoma,” npj Precision Oncology 5, no. 1 (2021): 10. 33580183 10.1038/s41698-021-00147-6 PMC7881100

[22] I. Porcellato , C. Brachelente , K. Cappelli , et al., “FoxP3, CTLA‐4, and IDO in Canine Melanocytic Tumors,” Veterinary Pathology 58, no. 1 (2021): 42–52. 33021155 10.1177/0300985820960131

[23] I. L. Bergin , R. C. Smedley , D. G. Esplin , W. L. Spangler , and M. Kiupel , “Prognostic Evaluation of Ki67 Threshold Value in Canine Oral Melanoma,” Veterinary Pathology 48, no. 1 (2011): 41–53. 21123859 10.1177/0300985810388947

[24] I. Porcellato , S. Silvestri , L. Menchetti , et al., “Tumour‐Infiltrating Lymphocytes in Canine Melanocytic Tumours: An Investigation on the Prognostic Role of CD3+ and CD20+ Lymphocytic Populations,” Veterinary and Comparative Oncology 18, no. 3 (2020): 370–380. 31750993 10.1111/vco.12556

[25] L. Aresu , L. Marconato , V. Martini , et al., “Prognostic Value of PD‐L1, PD‐1 and CD8A in Canine Diffuse Large B‐Cell Lymphoma Detected by RNAscope,” Veterinary Sciences 8, no. 7 (2021): 120. 34209830 10.3390/vetsci8070120 PMC8310184

[26] P. Bankhead , M. B. Loughrey , J. A. Fernández , et al., “QuPath: Open Source Software for Digital Pathology Image Analysis,” Scientific Reports 7, no. 1 (2017): 1–7. 29203879 10.1038/s41598-017-17204-5 PMC5715110

[27] I. Porcellato , M. Sforna , A. Lo Giudice , et al., “Tumor‐Associated Macrophages in Canine Oral and Cutaneous Melanomas and Melanocytomas: Phenotypic and Prognostic Assessment,” Frontiers in Veterinary Science 9 (2022): 878949. 35937296 10.3389/fvets.2022.878949 PMC9355725

[28] P. J. Bergman , “Canine Oral Melanoma,” Clinical Techniques in Small Animal Practice 22, no. 2 (2007): 55–60. 17591290 10.1053/j.ctsap.2007.03.004

[29] P. Bergman , E. Laura , and M. Kent , “Melanoma,” in Withrow and MacEwen's Small Animal Clinical Oncology, 6th ed., eds. D. M. Vail , D. Thamm , and J. Liptak (Philadelphia, PA: W.B. Saunders, 2019), 367–381.

[30] J. M. Placke , M. Kimmig , K. Griewank , et al., “Correlation of Tumor PD‐L1 Expression in Different Tissue Types and Outcome of PD‐1‐Based Immunotherapy in Metastatic Melanoma—Analysis of the DeCOG Prospective Multicenter Cohort Study ADOREG/TRIM,” eBioMedicine 96 (2023): 104774. 37660535 10.1016/j.ebiom.2023.104774 PMC10483509

[31] G. J. Kaunitz , T. R. Cottrell , M. Lilo , et al., “Melanoma Subtypes Demonstrate Distinct PD‐L1 Expression Profiles,” Laboratory Investigation 97, no. 9 (2017): 1063–1071. 28737763 10.1038/labinvest.2017.64 PMC5685163

[32] Y. Han , D. Liu , and L. Li , “PD‐1/PD‐L1 Pathway: Current Researches in Cancer,” American Journal of Cancer Research 10, no. 3 (2020): 727–742. 32266087 PMC7136921

[33] S. Chen , G. A. Crabill , T. S. Pritchard , et al., “Mechanisms Regulating PD‐L1 Expression on Tumor and Immune Cells,” Journal for Immunotherapy of Cancer 7, no. 1 (2019): 1–12. 31730010 10.1186/s40425-019-0770-2 PMC6858680

[34] R. Owaki , T. Deguchi , S. Konnai , et al., “Regulation of Programmed Death Ligand 1 Expression by Interferon‐γ and Tumour Necrosis Factor‐α in Canine Tumour Cell Lines,” Veterinary and Comparative Oncology 21, no. 2 (2023): 279–290. 36802270 10.1111/vco.12886

[35] N. Maekawa , S. Konnai , R. Ikebuchi , et al., “Expression of PD‐L1 on Canine Tumor Cells and Enhancement of IFN‐γ Production From Tumor‐Infiltrating Cells by PD‐L1 Blockade,” PLoS One 9, no. 6 (2014): e98415. 24915569 10.1371/journal.pone.0098415 PMC4051644

[36] J. Madore , D. Strbenac , R. Vilain , et al., “PD‐L1 Negative Status Is Associated With Lower Mutation Burden, Differential Expression of Immune‐Related Genes, and Worse Survival in Stage III Melanoma,” Clinical Cancer Research 22, no. 15 (2016): 3915–3923. 26960397 10.1158/1078-0432.CCR-15-1714

[37] J. M. Taube , A. Klein , J. R. Brahmer , et al., “Association of PD‐1, PD‐1 Ligands, and Other Features of the Tumor Immune Microenvironment With Response to Anti‐PD‐1 Therapy,” Clinical Cancer Research 20, no. 19 (2014): 5064–5074. 24714771 10.1158/1078-0432.CCR-13-3271 PMC4185001

[38] M. W. L. Teng , S. F. Ngiow , A. Ribas , and M. J. Smyth , “Classifying Cancers Basedon T‐Cell Infiltration and PD‐L1,” Cancer Research 75, no. 11 (2015): 2139–2145. 25977340 10.1158/0008-5472.CAN-15-0255 PMC4452411

[39] V. B. Stevenson , S. N. Perry , M. Todd , W. R. Huckle , and T. LeRoith , “PD‐1, PD‐L1, and PD‐L2 Gene Expression and Tumor Infiltrating Lymphocytes in Canine Melanoma,” Veterinary Pathology 58, no. 4 (2021): 692–698. 34169800 10.1177/03009858211011939

[40] S. Kleffel , C. Posch , S. R. Barthel , et al., “Melanoma Cell‐Intrinsic PD‐1 Receptor Functions Promote Tumor Growth,” Cell 162, no. 6 (2015): 1242–1256. 26359984 10.1016/j.cell.2015.08.052 PMC4700833

[41] M. Morsey , M. Stock , A. Boeckh , and H. Lehmann , “An Anti‐Canine PD‐1 Monoclonal Antibody for Immunotherapy of Cancer in Dogs. 2021 ACVIM Forum Research Abstract Program,” Journal of Veterinary Internal Medicine 35 (2021): 3020–3021.

[42] A. Giuliano , P. A. B. Pimentel , and R. S. Horta , “Checkpoint Inhibitors in Dogs: Are We There Yet?,” Cancers (Basel) 16, no. 11 (2024): 1–16. 10.3390/cancers16112003 PMC11171034 38893123

[43] T. Deguchi , N. Maekawa , S. Konnai , et al., “Enhanced Systemic Antitumour Immunity by Hypofractionated Radiotherapy and Anti‐PD‐L1 Therapy in Dogs With Pulmonary Metastatic Oral Malignant Melanoma,” Cancers (Basel) 15, no. 11 (2023): 4–15. 10.3390/cancers15113013 PMC10252299 37296981

[44] E. I. Buchbinder and A. Desai , “CTLA‐4 and PD‐1 Pathways Similarities, Differences, and Implications of Their Inhibition,” American Journal of Clinical Oncology: Cancer Clinical Trials 39, no. 1 (2016): 98–106. 10.1097/COC.0000000000000239 PMC4892769 26558876

[45] M. P. Pistillo , P. L. Tazzari , G. L. Palmisano , et al., “CTLA‐4 Is Not Restricted to the Lymphoid Cell Lineage and Can Function as a Target Molecule for Apoptosis Induction of Leukemic Cells,” Blood 101, no. 1 (2003): 202–209. 12393538 10.1182/blood-2002-06-1668

[46] S. Laurent , P. Queirolo , S. Boero , et al., “The Engagement of CTLA‐4 on Primary Melanoma Cell Lines Induces Antibody‐Dependent Cellular Cytotoxicity and TNF‐α Production,” Journal of Translational Medicine 11 (2013): 1–13. 23634660 10.1186/1479-5876-11-108 PMC3663700

[47] M. P. Pistillo , R. Carosio , F. Grillo , et al., “Phenotypic Characterization of Tumor CTLA‐4 Expression in Melanoma Tissues and Its Possible Role in Clinical Response to Ipilimumab,” Clinical Immunology 215 (2020): 108428. 32344017 10.1016/j.clim.2020.108428

[48] J. Yang , M. Dong , Y. Shui , et al., “A Pooled Analysis of the Prognostic Value of PD‐L1 in Melanoma: Evidence From 1062 Patients,” Cancer Cell International 20, no. 1 (2020): 1–11. 32256205 10.1186/s12935-020-01187-x PMC7106672

[49] A. Fanelli , L. Marconato , L. Licenziato , L. Minoli , N. Rouquet , and L. Aresu , “POT1 Mutations Are Frequent and Associated With Ki‐67 Index in Canine Diffuse Large B‐Cell Lymphoma,” Frontiers in Veterinary Science 9 (2022): 968807. 36016811 10.3389/fvets.2022.968807 PMC9396242

