# Supplementary material for: PD‐L1, PD‐1, and CTLA‐4 mRNA In Situ Expression by Canine Oral Melanoma Cells and Immune Cells of the Tumour Microenvironment
Source: Vet Comp Oncol. 2025 Jan 9;23(2):141–51. doi: 10.1111/vco.13039 (PMC12082799; doi:10.1111/vco.13039)
Supplement: Supplementary file 1 — Data S1. Supporting Information. [file VCO-23-141-s001.docx]

**Supplementary Table S1.** Signalment, localization, staging and therapy after surgery of canine oral melanoma cases included in the study.

| **Case N.** | **Breed** | **Age** | **Sex** | **Localization** | **Stage** | **Therapy** | **Progression** | **Disease Free Interval (days)** | **Overall Survival**  **(days)** | **Cause of death** |
| --- | --- | --- | --- | --- | --- | --- | --- | --- | --- | --- |
| 1 | German Shepherd | 10 | NF | mandible | III | immunotherapy^†^, chemotherapy^‡^ | yes (R, Met) | 61 | 227 | melanoma |
| 2 | Dachshund | 10 | M | mandible | III | chemotherapy | no | 1226 | 1226 | alive |
| 3 | Mixed breed | 10 | M | mandible | III | immunotherapy, chemotherapy | yes (Met) | 53 | 66 | melanoma |
| 4 | Golden Retriever | 12 | M | mandible | III | no | yes (Met) | 332 | 638 | abdominal abcess |
| 5 | Dachshund | 11 | NF | mandible | III | immunotherapy | yes (Met) | 131 | 902 | alive |
| 6 | Zwergschnauzer | 7.5 | M | maxilla | II | immunotherapy chemotherapy | yes (Met) | 261 | 452 | melanoma |
| 7 | Golden Retriever | 10.5 | NM | mandible | III | immunotherapy, chemotherapy | yes (Met) | 60 | 138 | multiple myeloma |
| 8 | Golden Retriever | 10 | NF | mandible | III | immunotherapy, chemotherapy | yes (R, Met) | 116 | 383 | melanoma |
| 9 | Belgian Malinois | 8 | NF | mandible | III | immunotherapy, chemotherapy | yes (Met) | 102 | 182 | melanoma |
| 10 | Labrador Retriever | 12 | NM | maxilla | II | chemotherapy | yes (R, Met) | 212 | 335 | melanoma |
| 11 | Mixed breed | 11 | NM | mandible | III | immunotherapy | yes (R, Met) | 256 | 598 | melanoma |
| 12 | German Shepherd | 10 | M | mandible | III | no | yes (Met) | 37 | 63 | melanoma |
| 13 | Airedale Terrier | 8 | F | maxilla, hard palate | II | chemotherapy | no | 710 | 710 | alive |
| 14 | Labrador Retriever | 9 | M | mandible | III | immunotherapy, chemotherapy | yes (Met) | 72 | 336 | melanoma |
| 15 | English Bulldog | 9 | M | cheek | III | immunotherapy, chemotherapy | yes (Met) | 74 | 171 | melanoma |
| 16 | Mixed breed | 12 | M | mandible | III | no | yes (R, Met) | 31 | 31 | melanoma |
| 17 | Mixed breed | 16 | NF | mandible | III | immunotherapy | yes (Met) | 28 | 94 | melanoma |
| 18 | Mixed breed | 15 | NM | lip | III | no | (Met) | * | * | melanoma |
| 19 | Beagle | 13 | M | cheek | III | no | yes (R) | n/a | 77 | melanoma |
| 20 | Mixed breed | 10 | F | oral mucosa | IV | electrochemotherapy | yes (R, Met) | 177 | 177 | melanoma |
| 21 | Mixed breed | 12 | F | lip | II | no | yes (R) | n/a | 1064 | melanoma |
| 22 | Golden Retriever | n/a | F | gingiva | IV | no | yes (Met) | n/a | 370 | melanoma |
| 23 | Mixed breed | 15 | M | oral mucosa | I | electrochemotherapy | no | 89 | 89 | bronchomalacia |
| 24 | Cocker Spaniel | 15 | F | oral mucosa | II | no | yes (R) | n/a | 96 | melanoma |

n/a = not available; F = female; NF = neutered female; M = male; NM = neutered male, R = recurrence, Met = metastasis.

^†^Immunotherapy was administered using an anti-chondroitin sulfate proteoglycan-4 (CSPG4) DNA-based vaccine, in all cases.

^‡^Chemotherapy was administered using a metronomic regimen, in all cases.

*Euthanized at the time of diagnosis and excluded from the survival analysis.

**Supplementary Figure S2.** Kaplan–Meier (KM) curves for overall survival (OS) of 23 dogs with canine oral melanoma. (A) KM curve of OS based on tumor infiltrating lymphocytes (TILs) grades. (B, C, D) KM curves of OS based on PD-L1, PD-1 and CTLA-4 ACD RNAscope^™^ scores, respectively.

**
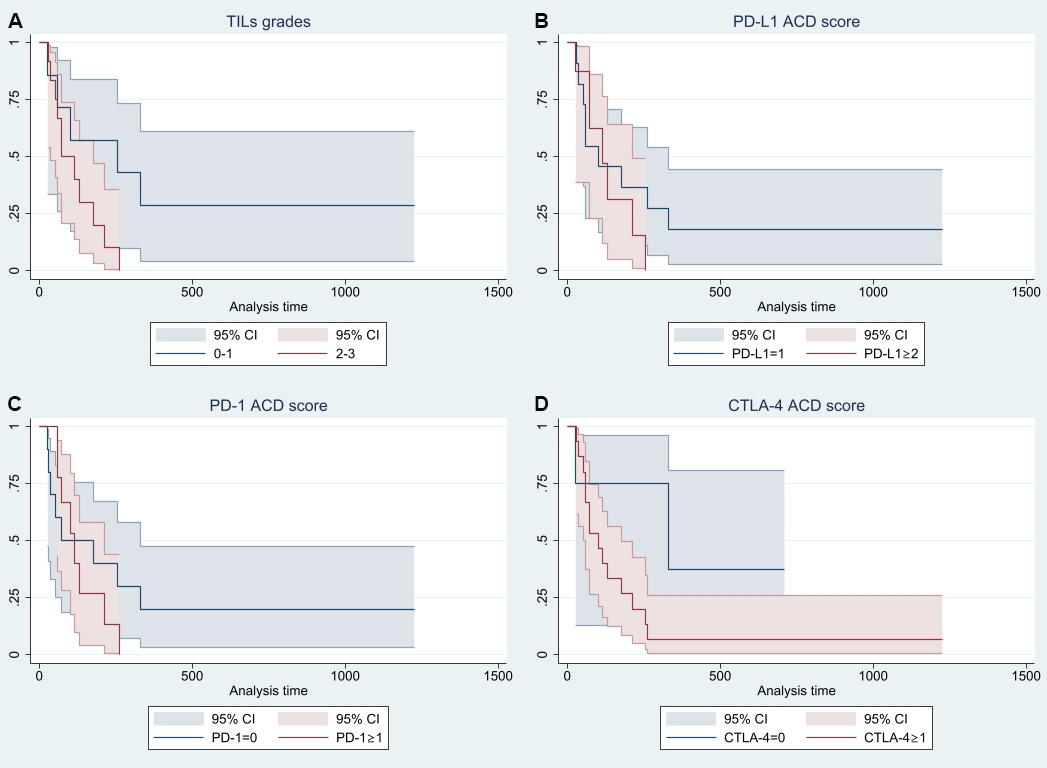
**

**Supplementary Figure S3.** Kaplan–Meier (KM) curves for disease free interval (DFI) of 19 dogs with canine oral melanoma. (A) KM curve of DFI based on tumor infiltrating lymphocytes (TILs) grades. (B, C, D) KM curves of DFI based on PD-L1, PD-1 and CTLA-4 ACD RNAscope™ scores, respectively.**
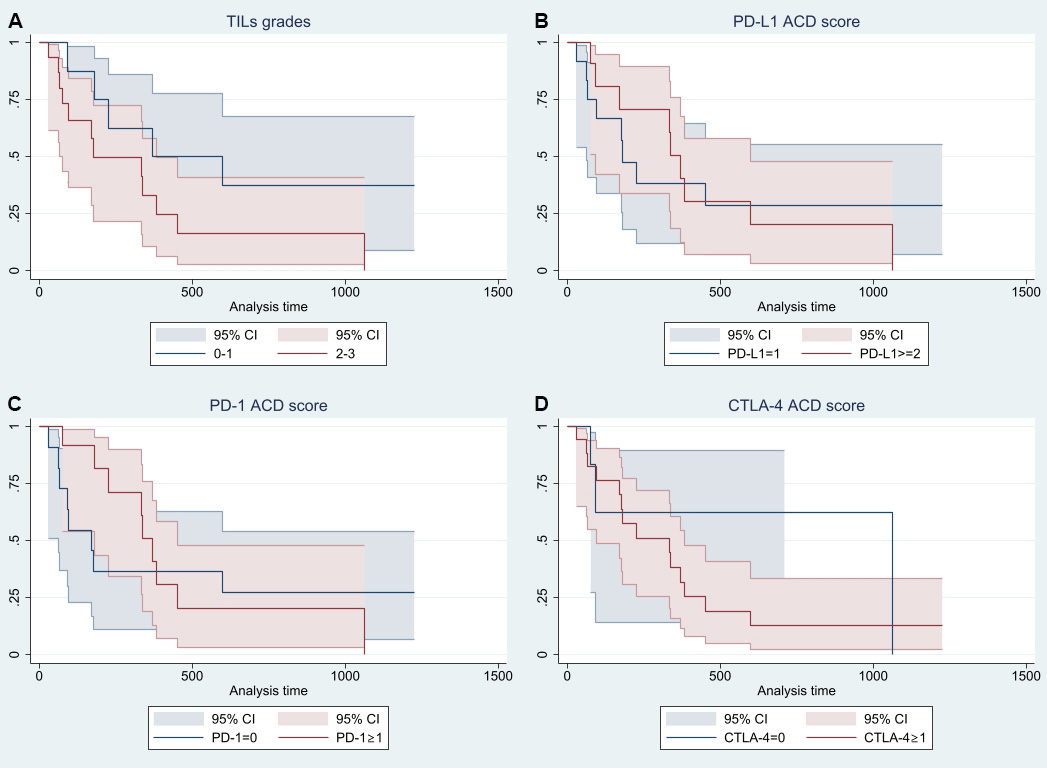
**
